# Clinical evaluation, accurate diagnosis and treatment of four pedigrees with Fabry's disease

**DOI:** 10.3389/fped.2023.1057014

**Published:** 2023-02-15

**Authors:** Peng Gou, Jie Leng, Xinran Cheng, Jing Zhang

**Affiliations:** Department of Pediatric Genetics, Endocrinology and Metabolism, Chengdu Women and Children's Central Hospital, School of Medicine, University of Electronic Science and Technology of China, Chengdu, China

**Keywords:** fabry's disease, children, genotype, family, enzyme replacement therapy

## Abstract

**Objective:**

This article analyzes the data of four families with mutations of the GLA (galactosidase) gene with a special focus on the clinical presentation, diagnosis, and interdisciplinary clinical management of Fabry disease (FD) and enzyme replacement therapy (ERT) treatment, and has the aim to assess more accurate prevention and treatment strategy.

**Methods:**

The MSSI (Mainz Severity Score Index) scale was used to evaluate the clinical data of five children diagnosed in our hospital, and the genotypes of all the patients with FD were collected. Two of the male children started ERT. We summarize the clinical effect and the evaluation of globotriaosylsphingosine (Lyso-GL-3) before and after treatment.

**Results:**

Five children were confirmed as having FD using the family histories, clinical manifestations, *α*-galactosidase A (a-Gal A) activity, and genetic test results. Two children used agalsidase *α* every 2 weeks regularly, after ERT. Their clinical symptoms improved, their pain intensity was significantly relieved, and upon re-examination their Lyso-GL-3 decreased conspicuously and no serious adverse reactions occurred. We report for the first time four families with children with FD. The youngest child was only 1 year old. The four families included one girl which is rare in X-linked lysosomal storage diseases.

**Conclusion:**

The clinical phenotype of FD in childhood is nonspecific, and the misdiagnosis rate is high. Most children with FD have a delayed diagnosis, and their organs are often seriously damaged in adulthood. Pediatricians must improve their diagnosis and treatment awareness, screen high-risk groups, and emphasize multidisciplinary cooperation and holistic lifestyle management after diagnosis. The diagnosis of the proband is also conducive to the mining of other cases of FD families and has important guiding significance for prenatal diagnosis.

## 1. Introduction

Fabry's disease (FD) is a rare X-linked lysosomal storage disorder. It is a variation of the *GLA* gene that causes the decrease in the activity of *α*-GalA, which leads to the accumulation of the metabolic substrate globotriaosylceramide (Gb3) and its derivative sphingoid base, Lyso-GL-3, in the lysosomes of virtually all cell types of the body, and finally leads to the dysfunction and organic lesions of the heart, kidney, nerves and other important organs. It is one of 121 rare diseases in a catalog issued in China in May, 2018 (1). As the course of FD is chronically progressive, it gradually leads to multiple organ failure and premature death. ERT has been used worldwide for 20 years as a major therapeutic scheme with definite efficacy (2). ERT can effectively clear the storage of Gb-3 in patients' organs, improve organ functions, reduce and reverse organ damage, and makes FD a kind of hereditary lysosomal storage disease that can be treated. Therefore, ERT is the main treatment strategy recommended by national guidelines (2, 3). FOS (4) research pointed out that the younger patients start ERT, the greater the benefit for patients. The common symptoms of classical FD in childhood are neuropathic pain, hypohidrosis or anhidrosis, and corneal whorls, which may be accompanied by growth restriction, delayed puberty development (5), proteinuria, etc. However, due to the nonspecific clinical phenotype, the degree of damage to important organs in children is less than that in adults (although the activity of *α*-GalA is reduced). The identification of children with FD is a challenge for pediatricians.

**Figure 1:**
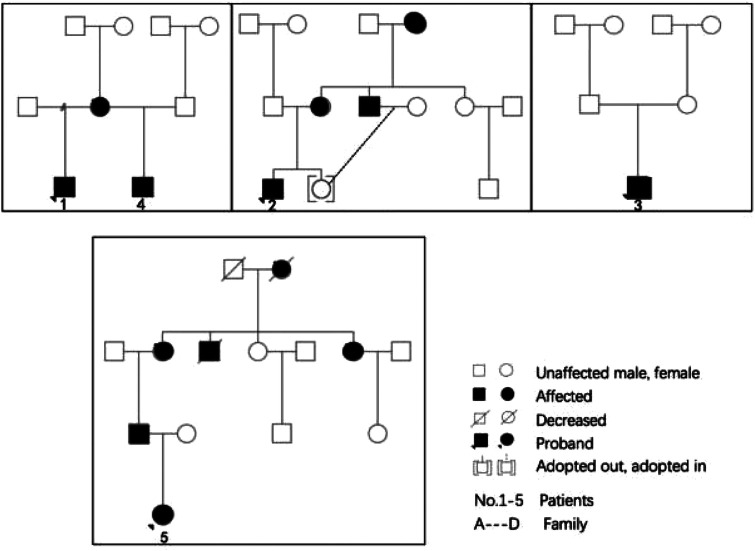
Family diagram of 5 children.

**Figure 2:**
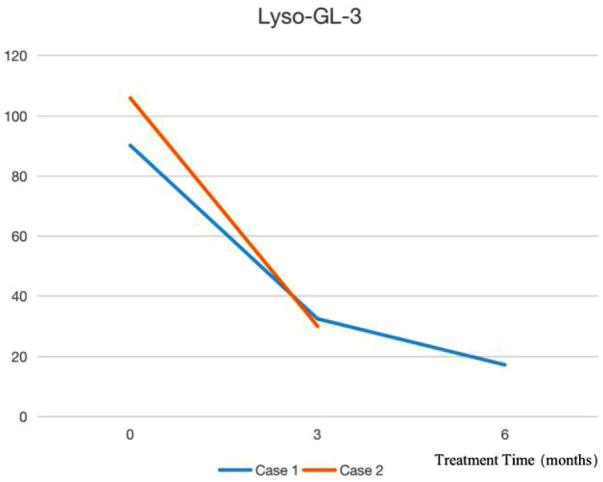
Indicators of lyso-GL-3 before and after treatment.

## 2. Objects and methods

### 2.1. Objects

From October 2021 to May 2022, five children with FD diagnosed in Chengdu Women's and Children's Central Hospital were selected as the study subjects. The study was approved by the Medical Ethics Committee of Chengdu Women's and Children's Central Hospital, and informed consent was exempted.

### 2.2. Methods

#### 2.2.1. Collection of clinical data

The outpatient and inpatient medical records of the children were checked through the hospital's electronic medical record system, and the clinical data were collected, including gender, age, height, weight, body mass index (BMI), puberty development stage, angiokeratoma, clinical phenotype, and time from symptoms to diagnosis. Furthermore, data from laboratory examinations including routine blood and urine examination, liver and renal function, blood glucose, serum lipid, electrolyte detection, and other laboratory examinations were collected. Finally, the clinical data also included hearing and fundus examination, pulmonary function testing, electrocardiograms, gastrointestinal electrograms, echocardiography, color doppler ultrasound of large blood vessels, chest CT scans, head MRI scans, *α*-GalA activity test, gene results, and Lyso-GL-3 test values before and after ERT.

#### 2.2.2. Diagnosis of classical FD

The clinical symptoms of children with classic FD are different from those of adults. Because the accumulation of metabolic substrates in important organs such as the heart, blood vessels, and kidneys are not serious in this period, hypertrophic cardiomyopathy, chronic renal dysfunction, and ischemic stroke are rare in children, while peripheral nerve symptoms such as acral burning pain, hypohidrosis or anhidrosis are common. Some children may experience growth restriction and delayed puberty development. Physical examinations for angiokeratoma of the skin, abnormal fundus, and urine should be performed. Its diagnosis needs to be combined with clinical symptoms, signs, and family history. According to a consensus of Chinese experts in the diagnosis and treatment of FD in 2021, the diagnosis should be confirmed through testing for the reduction of a-Gal A enzyme activity, the increase of metabolic substrate (Gb-3), and the variation of the *GLA* gene (6).

#### 2.2.3. ERT treatment

ERT treatment is the main treatment strategy recommended by international guidelines for children with FD. In 2001, two enzyme replacement therapies were approved by the European Medicines Agency (EMA) (1). Two forms of ERT drugs are used for lifelong treatment, with an intravenous injection once every two weeks. Agalsidase *α* (Replagal, Shire, Cambridge) at the dose of 0.2 mg/kg every two weeks is approved for children and adolescents aged 7 and above. Agalsidase *β* (Agalsidase, Sanofi Genzyme) at the dose of 1.0 mg/kg every other week was approved for children and adolescents. Agalsidase *α* was added to the National Healthcare Security Administration list in December 2021. Agalsidase *α* is a human protein produced by genetic engineering technology in human cell lines. The precise infusion tube with a built-in low protein binding filter (0.2 µm) is selected for infusion, the infusion rate is controlled at 15 mg/h, and the time of infusion should not be less than 40 min. According to the requirements of the drug instructions, this protocol meant that the possibility of transfusion-related reactions shall be minimized. In order to ensure the safety of ERT, antipyretic and first-aid drugs should be prepared regularly. Two children were treated with a 1-day hospitalization management mode, during ERT, ECG monitoring was conducted, and vital signs were closely monitored. Blood pressure was monitored 30 min after the start of the infusion, and the infusion time was approximately 40 min. Two of the children in the center did not have any adverse reactions to infusion. Due to the limitation of detection, anti—agalsidase alfa IgG antibodies were not detected.

### 2.3. Statistical analysis

Descriptive analysis. Counting data is represented by examples, and measurement data is represented by M (range).

## 3. Results

### 3.1. Basic information about the children

The clinical data of the five children are shown in Table 1. There were 4 male children and 1 female child. Their ages ranged from 1.1 to 13.1 years. Three patients were male children with significantly reduced enzyme activity. Case 1 was a 13.1-year-old male child who complained of short stature for 6 years, no puberty onset, and paroxysmal extremity pain. In October 2021 he was diagnosed with “short stature syndrome”. Finally, case 1 was diagnosed with FD through the discovery of a nonsense mutation of the *GLA* gene and low a-Gal A enzyme activity [0.36 umol/(L • h)]. Case 2 was a 10.5-year-old male. At the age of 6, there was no obvious onset, paroxysmal foot pain, and his mother and uncle were diagnosed as patients with FD. The same mutation site of the *GLA* was found in the child's gene test, and the a-Gal A enzyme activity was detected as low as [0.6 umol/(L • h)] to make a clear diagnosis. Case 3 was 1.1 years old and was the half-brother of case 1. The same *GLA* mutation site was found in the gene after case 1 was diagnosed, and the a-Gal A enzyme activity was low[0.35 umol/(L • h)]. The diagnosis age of case 4 was 1.4 years old. Due to being small-for-gestational-age and postnatal growth restriction, case 4 was diagnosed after next-generation sequencing. Case 5, a 7-year-old female, without clinical symptoms and with normal enzymology, was diagnosed because the child's genes were examined after her father's diagnosis.

**Table 1:** Clinical data of children with FD.

CASE	Gender	Age of diagnosis	Age of onset	BMI (kg/m^2^)	*α*-GalA μmol/ (L• h)	Lyso-GL-3 (ng/ml)	Site	Protein	Mutation type	Source
1	Male	13.1	5.0	12.75	0.36	90.21	c.1024C > T	p.R342*	Nonsense	Mother
2	Male	10.5	6.1	15.30	0.60	105.95	c.1025G > A	p.Arg342Gln	Missense	Mother
3	Male	1.1	–	18.75	0.35	39.64	c.1024C > T	p.R342*	Nonsense	Mother
4	Male	1.4	0.3	12.24	4.21	/	c.658C > T	p.Arg220*	Nonsense	De novo
5	Female	7.0	–	14.25	3.45	10.13	c.334C > T	p.Arg112Cys	Missense	Father

Note: α-GalA activity normal value 2.4–17.65 μmol/(L• h), Lyso-GL-3 normal value < 1.11(ng/ml).

### 3.2. Results of laboratory examination and auxiliary inspection

There were no left ventricular hypertrophies and the color doppler echocardiography showed the left ventricular ejection fractions were normal in the five children. The urine tests showed no proteinuria, normal creatinine range, no abnormal hearing (acoustic immittance or pure tone hearing threshold), and no abnormalities were found on the head MRI for the five children.

### 3.3. Evaluation of the clinical phenotype of FD patients with MSSI

The main manifestations of FD in childhood are neuropathic pain, proteinuria, and angiokeratoma. Table 2 shows the clinical phenotypes of the five children, evaluated with MSSI (7) scale from which the total score was obtained.

**Table 2:** FOS-MSSI (fabry outcome survey—mainz severity score Index).

Sign/symptom	General score				
	Case 1	Case 2	Case 3	Case 4	Case 5
Characteristic facial appearance	—	—	—	—	—
Angiokeratoma	1.5	0	0	0	0
Oedema	0	0	0	0	0
Musculoskeletal	1	1	1	0	0
Cornea verticillata	0	0	0	0	0
Diaphoresis	1	1	1	0	0
Abdominal pain	2	2	0	0	0
Diarrhea/constipation	0	0	0	0	0
Hemorrhoids	0	0	0	0	0
Pulmonary	0	0	0	0	0
New York Heart Association (NYHA) classification	0	0	0	0	0
Score	5.5	4	2	0	0
**Neurological score**					
Sign/symptom	Case 1	Case 2	Case 3	Case 4	Case 5
Tinnitus	0	0	0	0	0
Vertigo	0	0	0	0	0
Acroparaesthesia	4	6	0	0	0
Fever pain crisis	2	0	0	0	0
Cerebrovascular	0	0	0	0	0
Psychiatric/psychosocial	1	3	0	0	0
Depression	0	1	0	0	0
Fatigue	0	1	0	0	0
**Reduced activity**					
Level	0	1	0	0	0
Score	7	12	0	0	0
**Cardiovascular score**					
Sign/symptom	Case 1	Case 2	Case 3	Case 4	Case 5
Changes in cardiac muscle thickness	0	0	0	0	0
Valve insufficiency	0	0	0	0	0
ECG abnormalities	0	0	0	0	0
Pacemaker	0	0	0	0	0
Hypertension	0	0	0	0	0
Score	0	0	0	0	0
**Renal score**					
Sign/symptom	Case 1	Case 2	Case 3	Case 4	Case 5
Evidence of renal dysfunction	0	0	0	0	0
Score	0	0	0	0	0
**Total score**					
	Case 1	Case 2	Case 3	Case 4	Case 5
Total score	12.5	16	2	0	0

### 3.4. Gene detection and pedigree

Through gene testing, 4 mutation sites of *GLA* were found in the five children: c.1024 (exon7) C > T(p.R342*); c.1025 (exon7) G > A (p.Arg342Gln); c.658 (exon7) C > T p.Arg220*; and C334C > T (exon2) p.Arg112Cys. The mutation types include nonsense and missense mutations and the related mutation sites are reported in the literature. The mother of case 1 is a late-onset form FD patient, now 41 years old, whose a-Gal A enzyme activity was 0.76 umol/(L• h). No relevant abnormality was found in the other family members. Case 2 has a 26-year-old uncle, who is a classic FD patient with chronic renal insufficiency. The child's mother is 33 years old and is a late-onset form patient. At present, she still had acral pain, while other organs were not evaluated. His grandmother died at the age of 51 because of a “stroke”. Case 3 was the half-brother of case 1, the same GLA gene mutation site was found by gene detection. Case 4 was a *de novo* mutation. Case 5 was a late-onset form female patient and there are 6 patients with FD in the family.

There were five children with FD in our center from 4 families: 4 males and 1 female. The age ranged from 1.1 to 13.1 years old, including two cases of boys aged 1 to 2 years old. Case 1 was the first child diagnosed in our center, a 13-year-old boy who was found to have “Episodic feet pain for 8 years and growth retardation for 6 years”. Physical examination showed that his height and weight were all below the 3rd percentile of the same age and sex, and the testicular volume was 2 ml, Tanner I. When the first case was diagnosed, due to an insufficient understanding of the clinical symptoms of FD, the second-generation whole-exome sequencing showed that the *GLA* was a therapeutic mutation. We then completed the enzymology and Lyso-GL-3 examination. Case 2 was a 10.5-year-old boy. His mother and uncle were confirmed cases, and the child had intermittent and episodic foot pain for 5 years, and the diagnosis was confirmed by enzymology, and Lyso-GL-3 and gene examinations. Case 3 was the half-brother of Case 1, after the diagnosis of Case 1, *α*-GalA and genetic examinations were performed for Case 3. At the time of writing, he grows at a normal level for his age and gender, has no hypohidrosis, chronic diarrhea, vomiting, or other gastrointestinal symptoms, and no abnormalities in motor and language development. Case 4 was diagnosed at the age of 1.4 years because he was small for gestational age, and his postnatal growth restriction was confirmed after the second-generation sequencing was completed. Case 5 was the only female child in this group and was 7 years old. She had no clinical symptoms and normal enzymology but after her father's diagnosis, the child's genes and enzymology were examined and a diagnosis of FD was confirmed. The Family diagram of 5 children is shown in Figure 1.

### 3.5. ERT treatment status

After the diagnosis of Case 1, ERT treatment was started in March 2022, and the treatment was smooth for half a year. During the infusion process, his blood pressure was normal, without rash, angioneurotic edema, transient pain aggravation, or proteinuria (8). After 2 months of treatment, the patient's self-reported pain degree and attack frequency decreased significantly. Furthermore, his symptoms of hypohidrosis improved. After 3 months of treatment, Lyso-GL-3 was found to have decreased to 32.51 ng/ml from 90.21 ng/ml before infusion, and then to 17.2 ng/ml after 6 months of treatment. Case 2 started ERT treatment in April 2022 and the agalsidase *α* injection process was smooth. Two months later, the child's symptoms of hypohidrosis and acrodynia were significantly relieved, and he was willing to exercise. Three months later, Lyso-GL-3 was rechecked and had decreased from 105.95 ng/ml before the infusion to 30.04 ng/ml. No other organs were affected. Cases 3, 4, and 5 were not treated with ERT and were monitored regularly because Cases 3 and 4 were infants, and Case 5 had normal enzymology. Indicators of Lyso-GL-3 before and after treatment are shown in Figure 2.

## 4. Discuss

FD is a rare X-linked lysosomal storage disease, however, among lysosomal storage diseases, its morbidity is only second to Gaucher disease (9). Clinically, it has a chronic and progressive development. Due to the mutation of the *GLA* gene, *α*-GalA enzyme activity is partially or completely lost, resulting in its metabolic substrate Gb3 and the accumulation of its deacylated form Lyso-GL-3. This results in progressive renal failure, cardiomyopathy associated with cardiac arrhythmia, and recurrent cerebrovascular events, showing progressive functional impairment of varying degrees between individuals.

The mutations of the *GLA* gene, which is located on the Xq22 chromosome, have a length of 12 kb and consist of seven exons and six introns. More than 1,000 *GLA* mutations have been reported, and there is not a clear correlation between each genotype and clinical phenotype. Newborn screening for FD in Taiwan reveals higher incidences of 1:3,100 to 1:1,250 in males (10, 11). Mutations in our patients are mainly concentrated in exon 7, and also scattered in exon 2 and exon 5.

In recent years, with the progress of molecular diagnosis of diseases and the popularization of newborn screening, more hereditary diseases are diagnosed in infancy and childhood. FD is traditionally considered an adult disease, and the classic cases are mostly male. Although there is neuropathic pain, hypohidrosis, gastrointestinal discomfort, angiokeratoma, and other symptoms in childhood, the nonspecific symptoms often lead to a delayed diagnosis for decades. Therefore, the early diagnosis of FD, regular monitoring of confirmed cases in childhood, initiation of ERT treatment, and holistic lifestyle management can effectively prolong the life span and quality of life of patients. At the same time, the confirmed case, as the proband, is also very important for the determination of FD in the family and prenatal counseling after the determination of genotype.

The diagnosis of FD relies on the determination of *α*-GalA and Gb-3 in plasma or dried blood spots (12), and the *GLA* gene mutation. In recent years, Lyso-GL-3 has emerged as a novel, easily measurable marker as a pathogenic effector of FD. Compared with Gb3, it makes it easier to distinguish FD in non-classical types and non-patients, and it has become a widely used and accurate diagnostic tool for FD (13).

We report for the first time four families of children with FD, the youngest was only 1 year old. The four families included one girl, which is rare in X-linked lysosomal storage diseases. Family 1 contained two males and the sibling was born before the proband's diagnosis. The birth of Case 2 in Family 1 could have been avoided if prenatal diagnosis and identification of FD had been emphasized. It was reported that in males, a pathological low in leucocytes *α*-GLA activity indicates the presence of FD. In females, one of the two X chromosomes of each cell in the early embryo is randomly inactivated, so there is a high potential for residual enzymatic activity. The variability of the female clinical phenotype is greater than that of the male. Molecular genetic analysis is required for female diagnosis (14).

Among the cases reported by our center, Cases 1, 2, and 3 were classic FD patients. Cases 1 and 2 had clear symptoms of neuralgia and hypohidrosis. Case 1 also had obvious growth restriction and delayed puberty, which is similar to the phenotype of adolescent dysfunction reported in South Korea (5). In Case 3, *α*-GalA was significantly decreased, and Lyso-GL-3 was significantly increased, however, there is no obvious clinical phenotype at present, which is considered to be related to the patient's young age. Cases 4 and 5 were late-onset FD patients, showing a partial decrease in enzyme activity. The incidence of late-onset forms is more than 10 times that of the classic type (15), most of them occur in adults, and the symptoms are limited to the kidney or heart.

For the three children diagnosed by our center with classical FD, *α*-Gal A activity decreased significantly, and Lyso-GL-3 increased significantly. Compared with children with late-onset forms of FD, the concentration of the enzymological and metabolic substrates was significantly different. We observed that the clinical phenotypes of the children with the classic type were in the asymptomatic stage in infancy, but the concentration of Lyso-GL-3 in the dried blood spot on filter paper was 30 times higher than the normal value, confirming that the storage of metabolic substrates precedes the clinical phenotype. For the children with the classic type, the storage concentration of metabolic substrates increased with age. Spada (16) et al. also pointed out that the clinical phenotype of FD is caused by progressive GL-3 storage. Although there was no clinical phenotype and the enzymological index was normal in the two non-classical children, the Lyso-GL-3 index was at least 10 times higher than normal, which proved the diagnostic advantage of Lyso-GL-3 in differentiating non-classical patients from people without FD.

Three of the patients had nonsense mutations, one of which was a *de novo* mutation of somatic mosaicism with a mutation rate of 18%. Two cases were missense mutations. Although the four mutation sites have been reported (5, 17–19), there are few related studies, and no hot spot mutation has been suggested. Among them, the genotype of Case 4 was only reported in 1994, a 56-year-old Japanese woman with hypertrophic cardiomyopathy (19). The mutations of Cases 1, 2, and 3 were all from the mother. There were four adult patients in total including two families. It is worth noting that if Case 1 can be confirmed before case 3 is conceived by its mother, the mother would have received prenatal counseling when she conceived a second child, which could avoid the risk of delivering a child with FD. The mutation site for Case 5 was from her father. There were five adult patients in the family. In the above cases, the genotype of classic and non-classic patients contributed to organ function impairment in the adults, particularly damage of the heart and large blood vessels: valvular heart disease, hypertrophic cardiomyopathy, and hypertension. It was confirmed that the mutations of these four *GLA* gene loci were not functional mutations but pathogenic mutations (20). This proves that family screening is of great significance in finding new patients with FD. Based on the family screening of probands, an average of 3–5 new patients could be found (21). Because of X-linked inheritance, all the daughters of an affected father would inherit the mutation, while his son will not be affected. Of all the children of an affected mother, 50% would inherit the mutation. Therefore, genetic counseling should be recommended to all families with FD patients.

FD is a class of treatable hereditary lysosomal storage diseases. The goal of treatment is to delay disease progression, improve quality of life, reduce the incidence of related complications, and prolong survival. Disease management involves the assessment of organ function, monitoring of disease severity, enzyme replacement therapy, and treatment of complications. The treatment challenges faced by pediatricians for children with FD are pain management, treatment of gastrointestinal dysfunction, and the opportunity to start treatment. It is suggested that medical personnel with professional experience in chronic pain management should participate in the management of children's pain and skin hypohidrosis (3). In addition to preventing excessive skin temperature and guiding sports behavior, the available drugs include ibuprofen, gabapentin, and local drugs. In 2016, the consensus on the management and treatment of American children with FD in the USA was to give the infant or child doses of various drugs. The clinical manifestations of gastrointestinal dysfunction include abdominal pain, diarrhea, and vomiting. It is suggested that the treatment should be jointly managed with a gastroenterologist. PREDICT-FD points out that, in the opinion of the panelists, treatment should be initiated in any male patients with classical FD aged at least 16 years, and in younger males with classical FD if early signs of organ damage appear. Female patients and male patients with non-classical FD should be treated based on existing guideline recommendations (22) This is consistent with our practice of early initiation of ERT treatment in Cases 1 and 2.

ERT is the treatment strategy recommended by national guidelines. The current treatment options for FD include recombinant agalsidase *α* (0.2 mg/kg body weight) or agalsidase *β* (1 mg/kg body weight) every 2 weeks. Research shows that both drugs are safe for children to use. A large international retrospective study on the long-term outcome of FD in FOS was conducted with 677 patients in 24 countries over a time span of 15 years. The conclusion was that ERT provides long-term heart and kidney protection for patients, and ERT reduces the incidence of complex events in the heart, brain, or kidney, or even death. ERT treatment in childhood can prevent or delay organ damage. The younger the age of starting ERT, the greater the benefit to patients (23). Van (24) also argued that the early initiation of enzyme replacement therapy in male children with classic FD was associated with slower disease progression. PREDICT-FD reached a consensus that early indicators of renal damage included microalbuminuria, glomerular hyperfiltration, and podocyte inclusions in the presence of other renal lesions, such as signs of glomerulosclerosis or vasculopathy, which may occur even in patients without microalbuminuria (22).

In China, since December 2021, an agalsidase *α* injection has been included in the national medical insurance list, and the willingness for children to be diagnosed has greatly improved. The two patients with classic FD in our center, and their families, were fully informed before starting ERT treatment. For the evaluation, refer to 《The 2021 edition of the Expert Consensus on Diagnosis and Treatment of FD in China》. Both children reported significant improvement in extremity pain and hypohidrosis. Case 1 was treated for half a year, and the dried blood filter paper Lyso-GL-3 test results decreased by 80.9%. Three months after ERT treatment, the dried blood filter paper Lyso-GL-3 test results of Case 2 decreased by 71.64%. Later the detection of urine globotriaosylcera (25) could be carried out.

The future therapeutic approaches include next-generation ERT, substrate reduction therapy, and gene therapy. The long half-life (80 h) PEGylated [polyethylene glycol (PEG)] form of AGAL possibly allows the infusion interval (monthly intravenous administration) to be extended (26). Lucerastat (Idorsia) is a potential new oral treatment alternative. It has low molecular weight iminosugar and inhibits glucosylceramide synthase and the biosynthesis of glycosphingolipids, including upstream Gb3 (27). The principal aim of current gene therapy approaches in FD includes introducing DNA or RNA with the genetic code for the GLA gene into patients' cells. The aim is to further differentiate the transduced stem cells and express and secrete functional AGAL (23).

In the follow-up treatment, we will continue to pay attention to the symptoms of peripheral nerves, heart, and kidney function of the five children, and accurately detect the activity of *α*-Gal A enzymes, Lyso-GL-3, urine globotriaosylcera, and detect anti-agalsidase *α* IgG antibodies in children during ERT. In the follow-up treatment, we will continue to pay attention to the symptoms of peripheral nerves, heart and kidney function of 5 children, and accurately detect the activity of *α*-Gal A enzyme, dried blood filter paper Lyso-GL-3, urine globotriaosylcera, and simultaneous detection of anti-Agalsidase *α* IgG antibodies in children on ERT.

FD is a genetic lysosomal storage disease with an early definite cause (28), and it is also a kind of genetic disease with “target” drug treatment. In the field of pediatrics, more attention should be paid to prenatal diagnosis, neonatal gene screening, time to start ERT treatment, and interdisciplinary clinical management (29). At present, pediatricians' clinical awareness is very important due to the absence of neonatal gene screening for this disease. There is a question of whether there is a specific clinical phenotype for Chinese children. For example, Case 1 and Case 4 in this report have significant growth restrictions, and Case 1 had delayed puberty. The changes in substrate, antibodies and other indicators after the initiation of ERT treatment also require the accumulation of more cases.

## Data Availability

The original contributions presented in the study are included in the article/Supplementary Material, further inquiries can be directed to the corresponding author/s.
